# Molecular Chaperones, Cochaperones, and Ubiquitination/Deubiquitination System: Involvement in the Production of High Quality Spermatozoa

**DOI:** 10.1155/2014/561426

**Published:** 2014-06-19

**Authors:** Rosaria Meccariello, Rosanna Chianese, Vincenza Ciaramella, Silvia Fasano, Riccardo Pierantoni

**Affiliations:** ^1^Dipartimento di Scienze Motorie e del Benessere, Università di Napoli Parthenope, Via Medina 40, 80133 Napoli, Italy; ^2^Dipartimento di Medicina Sperimentale, Sezione “F. Bottazzi”, Seconda Università di Napoli, Via Costantinopoli 16, 80138 Napoli, Italy

## Abstract

Spermatogenesis is a complex process in which mitosis, meiosis, and cell differentiation events coexist. The need to guarantee the production of qualitatively functional spermatozoa has evolved into several control systems that check spermatogenesis progression/sperm maturation and tag aberrant gametes for degradation. In this review, we will focus on the importance of the evolutionarily conserved molecular pathways involving molecular chaperones belonging to the superfamily of heat shock proteins (HSPs), their cochaperones, and ubiquitination/deubiquitination system all over the spermatogenetic process. In this respect, we will discuss the conserved role played by the DNAJ protein Msj-1 (mouse sperm cell-specific DNAJ first homologue) and the deubiquitinating enzyme Ubpy (ubiquitin-specific processing protease-y) during the spermiogenesis in both mammals and nonmammalian vertebrates.

## 1. Introduction

Sexual reproduction is an evolutionarily conserved mechanism that guarantees genetic variability in order to preserve the biological biodiversity leading to differential survival of organisms within a population. It requires the production of highly specialized haploid cells, the gametes (spermatozoa in males and eggs in females) through the concerted occurrence of mitotic, meiotic, and differentiation events. In such a context, the control of protein folding and sorting is a fundamental checkpoint that guarantees the production of high quality gametes and the demolition of aberrant gametes. This subject matter appears more intriguing if analyzed from an evolutive point of view. The need to assure high quality spermatozoa production is surely shared not only by animal models that use mechanisms of external fertilization, such as fish and amphibian, but also by animals that use an internal fertilization strategy in which sperm cells undergo a complex set of transformations during the transit in male and female genital tracts (acquisition of motility in male reproductive tract and capacitation in female reproductive tract) in order to gain the full fertilizing ability. In this review, we will focus attention on male gametogenesis and point out the importance of molecular chaperones belonging to the superfamily of heat shock proteins (HSPs), their cochaperone DNAJ proteins, and ubiquitination/deubiquitination system in order to produce high quality spermatozoa. The evidences reported here come from nonmammalian vertebrate and mouse models, strongly supporting the existence of such evolutionarily highly conserved mechanisms to preserve gamete quality.

## 2. Involvement of HSPs, DNAJ Proteins, and Ubiquitination/Deubiquitination System in the Progression of Spermatogenesis and in the Production of High Quality Spermatozoa: An Overview

During spermatogenesis, the process that in males leads to the production of functional spermatozoa, a pool of stem cells named spermatogonia, has the ability to self-renew as well as to be committed, thus producing primary spermatocytes. These enter meiosis and produce in turn round spermatids and spherically symmetric haploid cells. Interestingly, a highly differentiation event, the spermiogenesis, consists in impressive morphological changes that allow the formation of spermatozoa. Proacrosomic vesicles formation, recognition, docking and fusion, sperm head elongation and transcriptional silencing due to chromatin remodeling, and the transient appearance of the microtubular manchette drive nuclear shaping [1], whereas giant mitochondrial and axoneme organization drive the formation of a flagellum [2]. Thus, the main changes that transform round spermatids into polarized spermatozoa, the cells that in orchestrated and finely modulated events gain the ability to reach and fertilize egg cell, require a deep regulation of cytoskeleton dynamics, vesicle trafficking, and protein sorting.

The complexity of the spermatogenesis is surely due to the coexistence of mitosis, meiosis, and cell differentiation in a unique process. Besides endocrine route—mainly orchestrated by the hypothalamic gonadotropin releasing hormone (GnRH), pituitary gonadotropins, and gonadal steroids [3]—there is an intragonadal network of regulators that allows intercellular-, intracellular-, and cellular-environmental communication [4–6]. Furthermore, a very hot topic concerns the involvement of HSP/DNAJ proteins [7] and ubiquitin/proteasome pathway [8] in the control of protein folding and sorting during the spermatogenesis.

**Figure 1:**
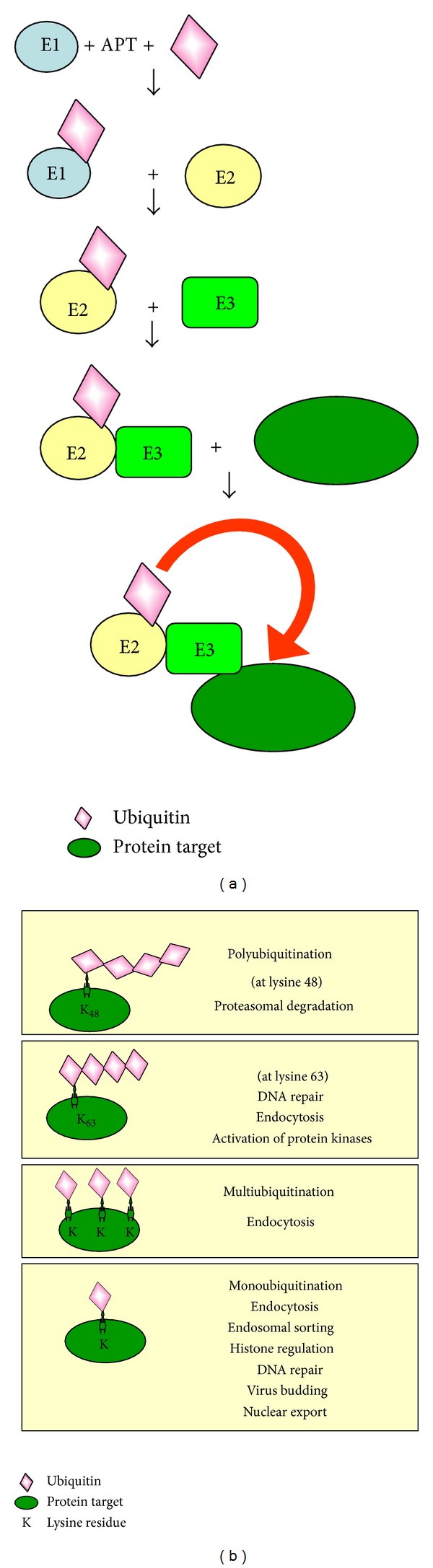
(a) The addition of ubiquitin at specific lysine residues on target protein requires the sequential activity of ubiquitin activating enzyme E1, ubiquitin conjugating enzyme E2, and ubiquitin ligase E3. (b) Different ubiquitin pathways: the addition of a chain of four ubiquitin molecules* via* lysine 48 in target protein is a tag for degradation into the proteasome; the addition of polyubiquitin chain* via* lysine 63 and multiubiquitin and monoubiquitin addition in target proteins activate cellular events other than proteasome dependent degradation (part of this figure was modified from [18]).

The members of 70 kDa HSP family (HSP70) are molecular chaperones able to regulate the folding, transport, and assembly of proteins into complexes [9], not only under stress conditions such as heat, but also under normal physiological conditions. They selectively bind the unfolded hydrophobic regions of substrate proteins and, by means of cycles of ATP binding, hydrolysis, and exchange, drive the correct protein folding [10]. The HSP70 activity requires the recruitment of molecular cochaperones belonging to the conserved family of HSP40/DNAJ proteins [11, 12]. All of them have just a highly conserved “J domain” by which they interact with HSP70, stimulating its ATPase activity (Type III DNAJ). Many DNAJ proteins have an additional Gly/Phe-rich region (Type II DNAJ) followed in the same cases by cysteine repeats (Type I DNAJ) [12]. HSP40/DNAJ proteins represent the largest HSP subfamily, at least in human where up to 41 different members have been identified [13]. Besides HSP70, also the family of 110, 60, and 90 kDa HSP (HSP110, 60, and 90, resp.) and the small HSP (sHSP) function as molecular chaperones being involved in processes such as the prevention of protein aggregation or the modulation of protein stability and conformation [14]. HSPs, HSP40/DNAJ proteins included, are conserved in both prokaryotes and eukaryotes [12, 14] and their impairment causes the accumulation of misfolded proteins that aggregate and cause cell damage or diseases such as neurodegenerative disorders and infertility in human [15–17]. The control of protein folding and sorting well correlates to the ubiquitination system, a conserved mechanism involved in the control of a set of proteolytical and nonproteolytical cell functions [18]. The ubiquitination system consists of the following components: (1) ubiquitin, a small heat stable protein of about 8.5 kDa [19] extensively found in a wide range of eukaryotic cells, but not in prokaryotic cells [20]; (2) ubiquitin activating enzyme E1; (3) ubiquitin conjugating enzyme E2; and (4) ubiquitin ligase E3. Ubiquitin covalently attaches to lysine residues of target protein but also has the ability to form polyubiquitin chains that are subsequently transferred to target protein. In this respect, the monoubiquitination is the addition of a single ubiquitin residue; the multiubiquitination is the addition of several single ubiquitin residues; lastly, the polyubiquitination is the addition of a polyubiquitin chain at specific lysine residues in target proteins [18]. Monoubiquitination, multiubiquitination, and polyubiquitination activate differential pathways leading to endocytosis, endosomal sorting, protein trafficking, histone regulation, DNA repair, nuclear export, cell cycle progression, cell proliferation/apoptosis, and proteolysis [18] (Figure 1). In case of protein misfolding, the polyubiquitination at lysine 48 is a tag for the proteasome pathway, one of the main processes of cellular protein degradation [8]. In this respect, the ability of deubiquitinating enzymes (DUBs) to edit the ubiquitination state of protein or to cleave polyubiquitin chains from substrates is a key step in the correct definition of tags for subcellular localization and intracellular trafficking of target protein [21].

In recent years, the relationship between these pathways and the spermatogenesis has been extensively studied [8, 22, 23] and the recent development of a “pharmacoperone” (pharmacological chaperone) based therapy [24] points out that these molecules may represent important pharmaceutical targets for the treatment of several human diseases, with infertility being included [24].

HSPs are widely expressed in the testes of several species and are deeply involved in the modulation of spermatogenesis and sperm functions [25]. In invertebrates, Hsp60C and Hsp60B are fundamental for spermatogenesis progression [26] and for spermatid individualization process in* Drosophila melanogaster* [27], whereas Hsp70 regulates spermatogenesis in the red claw crayfish* Cherax quadricarinatus* [28]. Hsp70 is involved in cellular remodeling processes and in the modulation of apoptosis rate in teleosts [29], a process also modulated by Hsp90 in newt spermatogonia [30]. Recently, fourteen members of HSP70 family have been identified in the genome of swamp eel,* Monopterus albus*, a freshwater natural sex-reversing fish, and the expression of one of them,* Hspa8b2,* was high, slight, and absent in testis, ovotestis, and ovary, respectively [31]. Two members of HSP70 family are specifically expressed in spermatogenic cells; in particular,* Hsp70-2* (currently known as* HspA2*) gene is expressed during the meiosis phase [32], while* Hsc70t* (currently known as* HspA1l*) is expressed during the postmeiotic phase [33]. Targeted mutation in* Hsp70-2* gene causes male infertility with massive apoptosis of pachytene spermatocytes and loss of spermatozoa [7]. Hsp70-2 is also associated with the synaptonemal complex; in fact, the lack of Hsp70-2 causes the missing separation (desynapses) of paired chromosome [34]. Germ cell apoptosis accomplished to* Hsp70-2* downregulation has also been reported in cases of testicular damage due to oxidative stress [35] and an unexpected role for Hsp70-2 in the control of spermatid DNA packaging proteins, the transition proteins 1 and 2, recently emerged [36]. Hsp70-2 is present in the acrosomal surface of human sperm and is impaired in idiopathic failure of sperm-egg recognition [37] as well as in infertile men with idiopathic oligoteratozoospermia [17]. In human, Hsp60 is expressed in both spermatogonia and ejaculated spermatozoa [38], whereas comparative immunolocalization of Hsp60, 70, and 90 has been provided in boar, stallion, dog, and cat spermatozoa providing evidence of species-specific activities related to fertilizing ability [39]. In rabbit, the distribution pattern of Hsp70 and Hsp90 was similar, both being mainly located in the spermatids of stage VII-VIII and in the cytoplasm of spermatogonia, whereas Hsp70-2 has been detected in the cytoplasm of pachytene spermatocytes and spermatids [40]. Lastly, Hsp90 is critical for the activation of the testis-specific serine/threonine kinases [41], for androgen receptor stability and functionality [42], and for meiotic progression of spermatocytes beyond pachytene stage [43]. The dynamic expression of HSPs during the spermatogenesis is upstream regulated by the heat shock transcription factors (HSF) [44], whose impairment has been linked to severe male reproductive defects [45, 46], including the pathogenesis of idiopathic azoospermia in human [47], a condition of severe male infertility due to unknown causes.

In testis, Hsp40/DNAJ proteins are involved in several processes, such as germ cells progression, apoptotic rate, androgen signaling, sperm tail formation, and acquisition of sperm function for fertilization as summarized in Table 1.

Apoptosis commonly occurs during spermatogenesis and is important to control the number of germ cells and to eliminate defective germ cells; the balance between germ cells division and germ cell loss is highly related to the ubiquitin-proteasome system [48]. Ubiquitination occurs from spermatocytes to spermatids and is finalized to high quality sperm production [8]. Accordingly, well-known dramatic changes occurring in spermatids are under the control of ubiquitination. One of them is the drastic reduction of cytoplasmic volume with half of mitochondria that are rejected in form of residual body. Thus, the colocalization of ubiquitin with mitochondria in spermatids and the ubiquitination of prohibitin—a mitochondrial inner membrane protein—seem to allow such a reduction [23]. Additionally, histone ubiquitination is an important instrument by which spermatids replace histones by the transition proteins and subsequently by protamines [49], and centrosome ubiquitination may support centrosome removal or reduction after it has fulfilled its role in generating sperm axoneme [23]. Moreover, some ubiquitination associated enzymes—such as E3 ubiquitin ligase—play an irreplaceable role in the formation of acrosome [8, 50–53]. In particular, the inactivation of* TMF/ARA160*, encoding a Golgi-associated protein that exhibits E3 ubiquitin ligase activity, results in the homing of Golgi-derived proacrosomal vesicles to the perinuclear surface; as a consequence, spermatozoa and epididymal sperm cells lack acrosome and present misshapen heads, tails coiling around the sperm heads, and lack of motility [50]. Similarly, the inactivation of the E3 ubiquitin ligase Cullin 4A (*Cul4*) causes male infertility as a consequence of decreased spermatozoa number, reduced sperm motility, and defective acrosome formation [51]. Interestingly, ubiquitination seems to be an important mechanism of sperm control also in the epididymis, where spermatozoa acquire the motility. In fact, ubiquitin is present in human seminal plasma [54], and defective spermatozoa in both humans and animals become ubiquitinated during epididymal passage to be then degraded by the proteasome [55].

Where there is ubiquitination, deubiquitination also occurs in a reversible manner. Two classes of deubiquitinating enzymes surely exert their activity at testicular level: (1) the ubiquitin-specific processing proteases (USP); (2) the ubiquitin C-terminal hydrolase (UCH). Besides the involvement in gonocyte recruitment and cell cycle progression [56], a growing body of evidences shows the involvement of DUBs especially during the progression into meiotic phase, the spermiogenesis, and the transit in the epididymis in order to direct the formation of high quality sperm and trigger apoptotic mechanisms that recognize and eliminate defective spermatozoa [23] (see Table 1 for details).

In such a context, the discovery of evolutionarily conserved molecular pathways under the control of HSP/DNAJ proteins and ubiquitin-proteasome/DUBs is intriguing and testifies that they play fundamental actions. Thus, in the next paragraph, we will discuss the conserved role played by the DNAJ protein, Msj-1 (mouse sperm cell-specific DNAJ first homologue, currently known as DnaJB3), and the DUB enzyme Ubpy (ubiquitin-specific processing protease-y), currently known as Usp8, during the spermiogenesis in both mammals and nonmammalian vertebrates.

## 3. Msj-1/Ubpy System and Acrosome Biogenesis

At the end of spermatogenesis, spermatids are subjected to structural modifications such as acrosome and tail formation. Such events are well known from a morphological point of view, but underlying signals and molecular mechanisms leading to them need to be better characterized. In particular, acrosome formation is a key event that has to be checked in order to produce sperm cells of good quality [57]. The acrosome is an acidic secretory vacuole critical for fertilization whose origin as direct Golgi-derived or lysosomal/endosomal secretory vesicle is still a matter of debate [58–60]. Biomarkers of both Golgian and plasma membrane/early endosome vesicles have been differentially identified during acrosome biogenesis [60–62], providing evidence that both membrane pathways may contribute to the formation of this testis-specific organelle. However, anterograde and retrograde trafficking pathways of proacrosomic vesicles are controlled step by step to ensure the right timing for fusion [59]. Such a coordinated process surely needs the participation of testis-specific modulators.

In this scenario, the DNAJ protein Msj-1 plays an important role [63, 64]. The* msj-1* gene was first isolated by a mouse spermatogenic cDNA library [65]. Interestingly, its transcript is specifically expressed in germ cells at haploid stages, as the protein appears in spermatids, especially in the periacrosomal and centriolar region in tight association with the testis-specific Hsp70-2 and the deubiquitinating enzyme Ubpy [63, 66, 67]. A deeper analysis at ultrastructural level reveals Msj-1 close to cellular membranous-vesicular system [68]. In particular, at the earlier phase of acrosome formation, the Golgi phase, a scattered Msj-1 immunolabelling marks the cytoplasmic area close to proacrosomic granules and Golgi apparatus. Then, as acrosome formation proceeds to the cap and acrosome phase, in the anterior part of the spermatids, Msj-1 labelling follows the contour of the developing acrosomic vesicles [66, 68]. Such studies of localization have prompted to speculate a possible role of Msj-1 in the regulation of acrosome formation. In order to confirm this hypothesis, wobbler mouse has been used as an experimental model [64]. This is a natural mutant characterized by motoneuron degeneration and defective spermiogenesis with sperm cells lacking a real acrosome and presenting an imperfect head position [69]. Both defects are due to a missense mutation (L967Q) affecting the gene that codifies Vps54, a vesicular sorting protein, component of Golgi associated retrograde protein (GARP) complex [70] that tethers vesicles from endosome to trans-Golgi network [71]. In particular, endoplasmic reticulum dilatation and abnormal protein accumulation in degenerating motoneurons [72] as well as missing of fusion of the proacrosomic vesicles during spermiogenesis [69] have been described in this mutant. Interestingly, both Msj-1 mRNA and protein have scantly been discovered in wobbler mouse testis, starting from 20* d.p.p*., when haploid spermatogenic stages appear [64]. Currently, the downregulation of Msj-1 expression observed in wobbler mouse seems to be combined to an alteration of testicular metabolism. In fact, the expression of *ER* *α* is also reduced as well as the intratesticular androgen content [64]; furthermore, mass spectrometric analysis reveals an altered concentration of protein associated with metabolite transport, fatty acid metabolism, cellular interactions, microtubule assembly, stress response, cell redox homeostasis, and detoxification [73]. Interestingly, both neurons and spermatozoa possess specialized vesicular organelles such as the neuronal signaling endosome and sperm acrosome, respectively, and both are polarized cells. Thus,* msj-1* mRNA has also been detected in the central nervous system, at ventral horn motoneuron levels [74], the other major site of cellular defect in the wobbler mice. In the cervical spinal motoneuron district,* msj-1* expression is significantly downregulated [74], as already observed in testis [64], thus suggesting that Msj-1 plays a central role in the defects regarding vesicle trafficking linked to the wobbler mutation.

Interestingly, Msj-1 has also been discovered in a lower vertebrate, the anuran amphibian,* Rana esculenta*, a seasonal breeder whose testis is progressively populated by germ cells at the same stage that develop in germinal cysts [75]. An expression analysis conducted during the annual sexual cycle revealed the presence of Msj-1 protein in isolated spermatozoa and in testis with a pattern closely associated with the end of meiosis and the onset of spermatid maturation [64, 76], exactly as observed in mice [63, 67]. Accordingly, an experiment of quiescence induction that causes the depletion of postmeiotic stages, decreases Msj-1 expression, completely confirming the presence of this protein in haploid germ cells [64]. What is sure is that the use of animal models phylogenetically distant is an important approach for detecting highly conserved molecules. This is the case of Msj-1 that may have a fundamental role during spermiogenesis, especially during acrosomogenesis, even if other functions related to protein folding and misfolding might be postulated.

Beyond Msj-1 expression in frog and mouse, nowadays blast search reveals the presence of* DnaJB3* gene homologue in human (ID: 414061), Norway rat (ID: 680216), crab-eating macaque (ID: 102124732), golden hamster (ID: 101841216), domestic ferret (ID: 101674438), European shrew (ID: 101557802), Southern white rhinoceros (ID: 101399203), Pacific walrus (ID: 101380753), Florida manatee (ID: 101353790), Western gorilla (ID: 101148022), Bolivian squirrel monkey (ID: 101053994), olive baboon (ID: 101009916), Northern white-cheeked gibbon (ID: 100594426), African savanna elephant (ID: 100669783), Rhesus monkey (ID: 100426730), and white-tufted-ear marmoset (ID: 100406349). Furthermore, in human genome database, two* msj-1* like genes have been described but only one gives rise to detectable mRNA [68, 77] and an Msj-1 like protein has been detected in human spermatozoa [68], suggesting a functional role, probably the same played in mouse and frog, in proper sperm functions. Interestingly, from a structural point of view, in both mouse and human,* msj-1* is an intronless gene located on chromosomes 1 and 2, respectively [68], in regions of demonstrated homology [78] containing the cluster of* UDP glucuronosyl-transferase (UGT) 1A* genes. The orientation of* Msj-1* gene is opposite to UGT family members and the gene is located into an intronic region of* UGT1A* genes. In mouse, antisense transcript for* msj-1* has been detected [79], thus not excluding the possibilities that nonfunctional gene discovered in human may exert a regulatory function.

A molecular partner of Msj-1 is the DUB enzyme Ubpy [67] that is highly expressed in brain and testis [80]. Ubpy is deeply involved in endosomal sorting, in vesicle trafficking events at the early to late endosome transition, and in the control of the number and size of endocytic vesicles [81–84] and for such a reason it has been considered a marker of acrosome biogenesis from the endocytic pathway. The gene is evolutionarily conserved being detected in primates (with human being included), oxen, rodents, monotreme mammals, birds, and amphibians and sharing a significant homology with DUB enzymes identified in echinoderms, insects, and fungi [85]. Since an* Msj-1* activity has been identified in amphibians [76], to verify how much conserved the Ubpy/Msj-1 system in vertebrates is, Ubpy synthesis has been checked in* R. esculenta* during the annual sexual cycle [86]. Its profile of expression, especially during November–May—a period in which massive spermiogenesis events occur—overlaps Msj-1 presence, previously described [76]. In addition, in November and July, Ubpy is located in round and elongating spermatids and spermatozoa, the same cellular types strongly immunopositive for Msj-1 [76], indicating the development of conserved functional roles.

Since* Ubpy* inactivation is lethal for the offspring [84], once again the use of mutant wobbler mouse provided insight to assess its role during the spermatogenesis.* Ubpy* expression has been analyzed in both normal and wobbler mice during the first wave of spermatogenesis together with the expression of* Hsp70-2* and* Hsp70t* [87]. Ubpy mRNA and protein were first detected at 10* d.p.p.* (appearance of preleptotene/leptotene spermatocytes) in line with previous reports concerning Ubpy localization in meiotic germ cells, spermatids, and spermatozoa [67]. Interestingly, in adult wobbler testis, where* Msj-1* expression is downregulated [64],* Ubpy* and* Hsp70t* mRNA are upregulated [87]. In addition, a differential sorting of Ubpy protein has been observed in spermatids of wobbler mice as compared with wild-type animals. In fact, while in wild-type testis Ubpy is mostly detected in soluble fraction, in wobbler testis it is primarily detected in membranous/insoluble protein fraction [87]. Furthermore, while in wild-type mouse Ubpy marks the surface of acrosomic vesicles, in wobbler mice—in which several acrosomic vesicles do not fuse in functional acrosome—only a detergent pretreatment allows to detect a diffuse and not polarized signal in the cytoplasmic/perinuclear area of round spermatids. From a morphofunctional point of view, while in wild-type testis several acrosomic vesicles expressing Vsp54 protein follow the route of Ubpy labeled vesicles [85], in wobbler mouse expressing a mutated Vsp54 protein, these vesicles are unable to coalesce into larger vesicles and both Vsp54 and Ubpy coated vesicles remain as scattered small vesicles into the cytoplasm [88]. Interestingly, Ubpy possesses a microtubule interacting and transport domain (MIT) [89] and* in vivo* it interacts with both spermatid endosomal sorting complex required for transport-0 (ESCRT-0) and microtubule structures [85]. In this respect, the current hypothesis postulates that in sperm cell Ubpy participates in acrosome biogenesis, acting as a linker between endosome pathway and microtubule cytoskeleton. In fact, in the early phase of acrosome biogenesis,* via* ESCRT-0 complex interaction, it recruits small vesicle protein to early endosome and directs the transport of ubiquitinated protein cargo in endosomal sorting through its MIT domain. Then, in participation with additional signals, such as Vsp54, Ubpy/ESCRT-0 tag may direct the vesicular cargo toward the proacrosomic vesicle, and, in the next step, when located on the acrosome surface, Ubpy could mediate the process of nuclear shaping interacting with the microtubule of the manchette complex.

The recruitment of additional partners in these routes has to be postulated, of course. However, these data surely highlight that Ubpy and Msj-1 work in concert to regulate basic activity during the spermatogenesis. Interestingly, such a speculation has been confirmed during evolution, thus suggesting a fundamental and conserved role played by this system.

## Figures and Tables

**Table 1:** 

Family	Name	Expression/localization and/or function	Species	References
DNAJ/HSP40	DnaJA1/DnaJA2	Spermiogenesis/androgen signaling	*Mus musculus *	[90]
	DnaJB1	Spermatocytes, round and elongating spermatids, sperm tail, and acrosome	*Mus musculus* *Rattus norvegicus*	[91]
	Msj-1 (DnaJB3)	Postmeiotic cells and spermatozoa, follows the contour of developing acrosome	*Mus musculus *	[63, 66]
	Mfsj-1	Spermatids	*Macaca fuscata *	[92]
	rDJL	Acrosome region of spermatozoa, participates in vesicular trafficking	*Rattus norvegicus *	[93]
	Tsarg-1/-3∗/-6	Inhibits spermatogenetic cell apoptosis	*Mus musculus* *Rattus norvegicus * *Homo sapiens *	[94] [95] [96, 97]
	DnaJB13	Axoneme formation	*Mus musculus *	[98]

USPs/UBPs	Usp2	Sperm motility and fertilization	*Mus musculus *	[99]
	Ubpy/Usp8	Acrosome biogenesis	*Mus musculus *	[67, 85, 87]
	Usp9Y	Male germ cell development	*Homo sapiens *	[100, 101]
	Usp14	Spermatid differentiation	*Mus musculus *	[102]
	Usp25	Testis	*Mus musculus *	[103]
	Usp26	Suggested role in sperm motility Blood-testis barrier and sperm head; regulator of germ-cell movement along the seminiferous epithelium	*Homo sapiens* *Mus musculus *	[104] [105]
	Usp42	Pachytene spermatocytes, round spermatids, and condensing spermatids	*Mus musculus *	[106]
	Usp44	Leydig cells and seminiferous epithelium	*Mus musculus *	[107]

UCHs	Uch-L1	Spermatogonia, Sertoli cells, caput epididymis, and vas deferens Mitotic proliferation, proapoptotic role during the progression of spermatogenesis and the transit in the epididymis	*Mus musculus *	[48, 108–110]
	Uch-L3	Meiotic pachytene spermatocytes and postmeiotic spermatids; cauda epididymis Sperm quality control during epididymal maturation	*Mus musculus *	[48, 109, 110]
	Uch-L4	All tissues, with testis included	*Mus musculus *	[111]
	Uch-L5	Spermatocytes and spermatids	*Mus musculus *	[112]
	CYLD	Control of spermatogenetic cell apoptosis and spermatogenesis progression via RIP1/NF-kappaB signalling axis	*Mus musculus *	[113]

*Indicates “also known as DnaJB13”.
